# Obesity in Children and the ‘Myth of Psychological Maladjustment’: Self-Esteem in the Spotlight

**DOI:** 10.1007/s13679-017-0246-y

**Published:** 2017-02-20

**Authors:** Andrew J. Hill

**Affiliations:** Academic Unit of Psychiatry and Behavioural Sciences Institute of Health Sciences, University of Leeds School of Medicine, Level 10, Worsley Building, Clarendon Way, Leeds, LS2 9NL UK

**Keywords:** Obesity, Children, Adolescents, Self-esteem, Victimisation, Peer relationships

## Abstract

**Purpose of Review:**

There are contrasting views regarding the psychological well-being of children with obesity. Responding to limitations of existing evidence, Jane Wardle in 2005 argued for a ‘myth of psychological maladjustment’. This review looks again at self-esteem.

**Recent Findings:**

The different characterisations of self-esteem each offer value. Global self-esteem is reduced in nearly all studies of youth with obesity. Dimensional self-esteem reveals physical appearance, athletic and social competence as the most affected areas, confirmed by research that has operationalised low self-competence. Children with obesity are also more likely to be victimised by their peers, generally and for their fatness. Victims who bully others appear to preserve some aspects of self-esteem.

**Summary:**

A relatively small proportion of youth with obesity has low self-esteem, but those with severe and persistent obesity are especially compromised. Weight loss is only weakly associated with improved self-competence suggesting the value of resilience and asset approaches to improving well-being.

## Introduction

One of Jane Wardle’s early interests was in children with obesity, their self-perception and self-esteem [1, 2]. This was commensurate with her broader regard for the needs of people with obesity, seen in her driving the establishment of the charity Weight Concern. At that time, I was also working and publishing on the self-perception of children with obesity and have continued this interest (while Jane’s research interests and outputs proliferated). One paper that stands out for me, and that I see regularly cited by others, was written by Jane with Lucy Cooke in 2005 [3]. If the title was benign, ‘The impact of obesity on psychological well-being’, the conclusion was not. They wrote ‘The persistence of the myth of psychological maladjustment of overweight and obese children is striking’.

The paper was a review of recent publications on body dissatisfaction, self-esteem and depression in children and adolescents with obesity. Interestingly, in the same year, Carl-Erik Flodmark published a short overview of the literature titled ‘The happy obese child’ [4]. Both publications shared the message that outside of a clinical environment, very few children with obesity are either depressed or have low self-esteem. Neither of these publications sought to dismiss children with obesity who are in distress and in need of help. Rather, they challenged practitioners to look again at the children they work with, not to generalise from extreme clinical experiences, to put aside preconceptions and to identify factors that protect psychological well-being.

The literature on self-esteem in childhood obesity exemplifies the challenge of understanding the psychology of young people with obesity. In their review, Wardle and Cooke noted the following problems [3]. Self-esteem appears poorer in clinical samples of youngsters with obesity than those from the community; so, it is unwise to generalise. Researchers rarely look beyond mean scores on self-esteem measures, or whether small differences in mean scores between children of healthy weight and those with obesity have real-life significance. Little effort has been directed to potential moderators or mediators of the relationship between obesity and self-esteem. Over the course of treatment, weight loss appears poorly related to any change in self-esteem. I would add that there has been little serious consideration of how self-esteem is conceptualised (and measured) in the context of obesity. Few authors have sought to define (and measure) *low* self-esteem and apply this to obesity. Furthermore, the predominant view of the relationship between obesity in childhood and self-esteem has been unidirectional rather than dynamic. Recognising the challenge laid down by Jane (and Lucy), a re-evaluation of the literature on self-esteem in children and adolescents with obesity is timely.

## Conceptualising Self-Esteem

Self-esteem is a long established psychological construct with a huge attendant literature. Self-esteem refers to the way that people perceive and value themselves. In more elaborated form it is, ‘the extent to which a person believes himself to be capable, significant, successful and worthy’ [5]. As Emler notes in his hugely influential review, the public discourse about self-esteem has moved forward [6]. In current usage, self-esteem is about psychological health and identity. It is a resource and an asset. High self-esteem is something we should have by right as it is good for the individual and for society.

In terms of how self-esteem is assessed, then a distinction can be made between self-esteem as a generalised or global self-appraisal, as competence in externally (and internally) valued domains and as a metric of social acceptance (or likely rejection). These perspectives each have something to say about the relationship between obesity and self-esteem.

### Global Self-Esteem

The idea that self-esteem can be assessed as an evaluative attitude to the self has been attributed to Rosenberg and his scale is regarded as the gold standard in self-esteem research [7]. The 10-item Rosenberg self-esteem scale concerns very general evaluations of oneself and yields a single score, a sum of positive statements. Its popularity is in part due to its simplicity and brevity.

Unsurprisingly, this scale is prominent in obesity research. In a meta-analysis looking at global self-esteem in all age groups, Miller and Downey found an effect size of −0.36 (95% CI −0.33 to −0.40), a robust but small to moderate sized relationship [8]. This confirms the difference in global self-esteem scores between people of healthy weight, who are overweight, and with obesity.

Important influences on the strength of this relationship were age and gender. The correlation between weight and self-esteem increased from −0.12 to −0.22 and −0.28, in children, adolescents and young college-age adults, respectively. In addition, the relationship was stronger in females (−0.23) than males (−0.09). More recently, a systematic review of studies comparing youth with obesity and healthy weight controls found lower global self-esteem scores in those with obesity in 17 of the 21 included studies [9•]. The four exceptions had a feature in common. They all reported on non-white ethnic groups: either samples from Asia or minority ethnic groups in the USA. The review authors urged caution, however, noting that there are other studies of youth and adults from the same countries and ethnicity/income groups that do show lower self-esteem in individuals with obesity [10].

### Perceived Self-Competence

The global perspective of self-esteem is in fact pre-dated by an elaborated conceptualisation. The representation of self-esteem as the ratio of a person’s successes to their pretensions has been attributed to William James [6]. Here, self-esteem is a personal evaluation of competence in areas viewed as important. So, there are two parts to this formulation of self-esteem: multiple domains in which the self is evaluated and a likelihood that some domains are more important than others. Indeed, it is the discrepancy between competence and importance that defines overall self-worth. Only when a person feels low competence in an area of high importance is their overall self-worth jeopardised.

There are only a handful of commonly used multidimensional measures of self-esteem for children and adolescents [11]. It is Susan Harter who has done most to develop the Jamesian conceptualisation and assessment of perceived self-competence [12]. She argues that for children, the necessary domains of competence are set by parents (scholastic competence and behavioural conduct) and peers (physical appearance, social and athletic competence). These domains expand in range through adolescence into adulthood, incorporating attributes such as job competence, romantic appeal and a sense of humour.

We conducted a systematic review of multi-competence assessments in young people with defined obesity. Studies that had only looked at overweight were excluded as we were interested in what the literature had to say specifically about the self-competence of those with obesity. There were 17 studies, of which 9 were cross-sectional and 7 weight management interventions [13]. Most had used Harter’s questionnaires. All of the studies that assessed physical appearance and athletic/physical competence found lower scores in youth with obesity. Obesity also impacted on perceived social acceptance, with lower scores reported in half of those measuring this domain. In contrast, few differences were observed in scholastic competence or behavioural conduct. Global self-worth was lower in children with obesity compared with those healthy weight in six of the nine cross-sectional studies, a finding comparable to that of the global self-esteem literature above. There were insufficient studies to detect any effects of age or sex. Likewise, comparisons based on race or ethnicity are infrequent in this literature. But the observation that in younger (9–12-year olds) minority children from low-income families, all, regardless of their weight status, had lower global self-worth than a reference white population [14] is a reminder of the inherent complexities in this area.

Thus far, this literature says much more about successes than pretensions in children with obesity. The competencies included in Harter’s self-perception profiles may indeed be those most important to today’s youth. Harter herself has written about how perceived physical appearance is the number 1 predictor of global self-worth [15]. This is true from age 5 through to adulthood. It raises the issue of how to help children value competencies other than appearance. But given that one way of managing poor competence is to diminish the importance of that feature, it is surprising that perceived importance has not been more thoroughly investigated. An assessment of domain importance is included in the manuals for Harter’s scales but rarely used in research. Our own unpublished work suggests that for a community sample of 12-year olds at least, healthy weight children and those with obesity do not differ in how important they rate appearance and athletic competencies. However, and in accord with the evidence presented above, they do perceive themselves very differently on these features.

#### Low Self-Esteem

The response format of the Harter measures permits one further and rarely reported feature of self-esteem: the assessment of low self-esteem. As acknowledged above, previous attention has focused on mean scale values that are statistically different but of questionable functional difference. Children completing Harter’s assessments go through a two-stage process in answering each question. The first requires them to identify with either a high or low self-competence characterisation. The second asks whether this description is ‘sort of’ or ‘really’ true for them. By setting a scale value at the point where a child indicates that they are like to the low competence description, then the proportions of low (and high) competence children can be compared across weight groups.

In a state-wide survey of 9–13-year olds from New South Wales, Australia, we found that perception of physical appearance was particularly affected, with 63% of girls and 33% of boys with obesity identifying with the depiction of a physically unattractive child [16]. In contrast, the proportion of low scorers on the global measure of self-worth was smaller. Although the relative risk of low global self-worth in girls with obesity was 4.1 times more than normal weight peers, only 20% of the group scored in this range. Complementing this, girls with obesity were more than five times *less* likely to have high global self-worth, something achieved by around 70% of their peers.

Danielsen et al., using the same approach to defining low self-esteem, also found higher proportions of Norwegian 10–13-year olds who were overweight/obese to have low physical appearance and athletic competence [17•]. For this population sample, the difference from healthy weight children extended to low social acceptance and scholastic competence, although the proportions were smaller than observed in the Australian children.

### The Looking-Glass Self

This rather different framing of self-esteem is attributed to Charles Cooley, and again, it is long-standing and highly influential. Its basis is that our assessments of our own worth are based on the judgements we imagine others make of us [6]. Moreover, our predictions about these judgements depend upon the qualities we see in these other people. So, what shapes self-esteem are not our accomplishments objectively and directly appraised, but the anticipated judgements of these accomplishments by other people. Hence, self-esteem is what we expect will be reflected by this social mirror, and the intensity of reflection depends on who we choose as our social referents.

Mark Leary has taken this social view in a particular direction, one very relevant to obesity. Sociometer theory proposes that the self-esteem system evolved primarily as a monitor of social acceptance, the motivation being not to maintain self-esteem *per se*, but to avoid social devaluation and rejection [18]. He argues that people are particularly sensitive to changes in relational evaluation or the degree to which others regard their relationship with the individual as valuable, important or close. Accordingly, self-esteem is lowered by failure, criticism or rejection and raised by success, praise and events associated with relational appreciation. Even the possibility of rejection can lower self-esteem. Two areas of research are particularly relevant to youth with obesity—interpersonal relations and victimisation.

#### Interpersonal Relations

Sociometric procedures using peer-nominated friendships have shown little impact of being obese in community samples of primary school aged children. Some 20 years ago, for example, young children with obesity in the UK were just as likely to be chosen as their lean peers as people to socialise with both inside and outside of school, even though they were judged as less attractive [19]. The situation is likely to be different now, as has been observed in the USA. In a very large community sample of 6–7-year olds, Harrist et al. used most and least liked peer nominations to generate standard social preference classifications [20•]. Children with obesity were more likely than healthy weight children to be neglected, i.e. with few positive or negative nominations. Those with severe obesity were significantly more likely to be rejected, i.e. with more least-liked, negative nominations. Even so, more than twice as many 6–7-year olds with obesity were classified as popular or average than were those rejected, neglected or controversial.

Looking at an older age group, data from the US National Longitudinal Study of Adolescent Health (Add Health) shows overweight adolescents to be over-represented in categories of no or few peer friendship nominations and under-represented in the most popular categories [21]. Most importantly, they received fewer reciprocal nominations: that is, nominations by peers they themselves had nominated. Further analysis of this cohort indicated that overweight adolescents whose friendship attempts with non-overweight peers were not reciprocated would turn to other overweight peers [22•]. Accordingly, in another and smaller sample of US teenagers, friendship choices showed that overweight youth were twice as likely to have overweight friends as their non-overweight peers [23].

The relative failure to be named a friend by people you nominate suggests that the friendship ties of adolescents with obesity are less plentiful, potentially weaker and more directed to others with obesity. In terms of self-esteem, the peer referent for self-evaluation chosen by teenagers with obesity determines their social standing: valued and held in esteem by others similarly overweight but likely rejected and so of low self-esteem in the eyes of those of healthy weight.

#### Victimisation

Peer difficulties and rejection have been observed in young children with obesity. By age 5, parents of children with obesity are more likely to report peer relationship problems in their girls and boys than parents of healthy weight children [24•]. Five-year olds themselves reject story characters drawn as fat as people they would choose to be friends with [25•]. Rejection may be a very small step from perceived victimisation.

The research evidence is unequivocal regarding the association between obesity and victimisation. A meta-analysis of 16 studies and 28 effect sizes showed a significant relationship between being obese and being victimised (OR = 1.51 (1.32, 1.71)) [26•]. Most of these studies were of children aged 11 and upwards. In an interesting development, observations by Primary school teachers in the Netherlands and the children themselves revealed that children with obesity were more likely to be victimised by their peers but also more likely to bully others [27•]. Indeed, there was a small group of children referred to as bully-victims who were both recipients and perpetrators of victimisation. Children with obesity were twice as likely to be in this category as healthy weight peers.

The work above has examined the generalised experience of victimisation without focusing on the reason for victimisation. Relatively, little work has looked specifically at weight-related victimisation in young people. We reported that some 42% of 9–12-year olds with obesity identified themselves as fat victimised compared with 7% of their healthy weight peers [28, 29]. Interestingly, being fat victimised was strongly associated with being victimised generally. In other words, those fat victimised were often those who felt victimised for other reasons too.

Of the sample of 815 English Primary and Secondary school children in these studies (440 boys, 375 girls; mean age = 11.0 years, range 9.0–12.6), 97 (11.9%) identified themselves as fat victimised and 42 (5.2%) as fat bullies. The assessment of fat victimisation was incorporated into Harter’s Self-Perception Profile for Children using two scales directed at victimisation and bullying developed by Austin and Joseph [30]. We retained three items from each scale and adapted items to form two new scales specific to fat victimisation and bullying. The result was a fat victimisation scale (e.g. some children are often bullied for being fat), a fat bully scale (some children often tease other children about being fat) and two scales in which the reason for victimisation was unspecified (e.g. some children are often called horrible names by other children). Internal reliability of these scales was good (α = 0.72 to 0.82) and did not vary by sex or age of respondents.

**Fig. 1:**
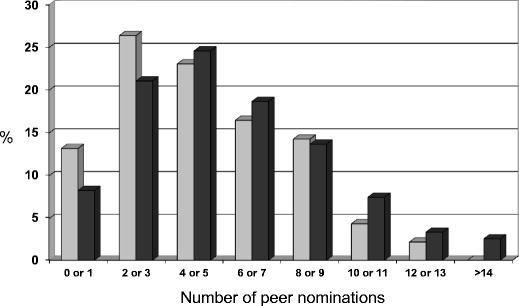
Peer popularity nominations received by fat-victimised (*light columns*) and non-victimised (*dark*) children. Previously unpublished results from [28, 29]

Fat victimised children were heavier and had a greater BMI *z*-score than those not victimised. They had significantly greater body dissatisfaction, and fat victimisation was strongly associated with current dieting to lose weight, with 35.1% of those victimised currently dieting compared with 11.6% of non-victimised children. Looking at their perceived self-competence, children fat victimised scored significantly lower on all domains, including global self-worth. Lower perceived social acceptance was congruent with significantly lower peer-nominated popularity (being nominated as someone that classmates would most like to sit next to in class and/or be with at breaktime), although the mean difference amounted to less than a half nomination per child (2.30 vs 2.81). Figure 1 shows that fat victimised children were more likely to receive very few peer nominations and less likely than non-victimised children to be nominated by many peers.

Of the 97 children fat victimised, 19 (13 boys, 6 girls) fat bullied others. A further 23 (21 boys, 2 girls) reported themselves as fat bullies without being victimised. There were more children with obesity in the bully-victim group (32%) than in the fat victimised (29%), fat bullies (18%) or not involved group (5%). This is consistent with the study of children in the Netherlands [27•]. However, it was those fat victimised whose risk of low domain competence was greatest. Table 1 shows that every domain was affected other than behavioural conduct (primarily at school). The relative risk of low global self-worth in fat victimised children was 5.39 (95% CI 3.40, 8.56). In contrast, fat bullies were compromised only in terms of their poor behavioural conduct. Looking at perceived high domain competence, then the bully-victim group shared with those fat victimised a failure to match their peer groups’ proportions of high scholastic competence, social acceptance and global self-worth.

**Table 1:** Relative risk (95% CI) of low and high perceived domain competence and global self-worth in fat victimized and fat bullying children (compared with the 695 ‘not involved’ children)

	Fat victimised	Fat bullies	Both fat victims and bullies
(N)	(78)	(23)	(19)
Low perceived competence			
Scholastic competence	**1.74** (**1.11**, **2.72**)	0.89 (0.34, 2.88)	1.19 (0.42, 3.43)
Social acceptance	**2.97** (**1.92**, **4.69**)	0.23 (0.01, 3.58)	2.32 (0.94, 5.72)
Athletic competence	**2.45** (**1.72**, **3.50**)	0.62 (0.16, 2.55)	1.12 (0.39, 3.21)
Physical appearance	**2.85** (**2.07**, **3.93**)	0.91 (0.31, 2.64)	1.82 (0.84, 3.97)
Behavioural conduct	1.46 (0.78, 2.73)	**3.96** (**2.16**, **7.28**)	**3.60** (**1.78**, **7.27**)
Global self-worth	**5.39** (**3.40**, **8.56**)	0.80 (0.11, 5.54)	2.89 (0.98, 8.53)
High perceived competence			
Scholastic competence	**0.65** (**0.45**, **0.93**)	0.70 (0.37, 1.30)	**0.06** (**0.00**, **0.89)**
Social acceptance	**0.68 (0.52, 0.89)**	0.91 (0.63, 1.31)	**0.42 (0.20, 0.90)**
Athletic competence	**0.40 (0.26, 0.63)**	1.11 (0.77, 1.60)	0.62 (0.32, 1.21)
Physical appearance	**0.42 (0.26, 0.66)**	1.22 (0.85, 1.76)	0.57 (0.27, 1.21)
Behavioural conduct	0.79 (0.60, 1.04)	**0.17 (0.04, 0.63)**	0.51 (0.24, 1.08)
Global self-worth	**0.51 (0.37, 0.70)**	0.79 (0.53, 1.18)	**0.32 (0.13, 0.77)**

Previously unpublished results from [28, 29]

Note: odds [95% CI] in bold are statistically significant

Three additional points are noteworthy. First, while being fat-teased was more common in children with obesity, over half did not identify themselves as such. We know very little about what has protected these children or what made the other half vulnerable. Second, our assessment of fat victimisation was directed at overt rather than relational victimisation, something consistent with the preponderance of boys in the bully group. Relational victimisation is more difficult to capture in questionnaires but may be extremely important in assessment of the true extent and consequences of fat victimisation. Third, victimisation did not impact on the perceived importance of any of these domains. Once more, it would appear that these children were not managing their low self-esteem by modifying the importance of domains in which they judged themselves less competent. Perhaps for these pre-teenagers, the possibility of diminishing the importance of such core areas in their lives is beyond imagination. They are just too young at this age to contemplate this.

### Consequences of Weight Management

In a review of the literature on structured weight management programmes for children and adolescents that included a measure of self-esteem, 18 of 21 studies were observed to report some end of intervention improvement in self-esteem [31]. This improvement appeared related to the following intervention components: consistent parental involvement, group-based interventions and actual weight loss.

We have previously noted the inconsistencies in associations between weight loss and self-esteem improvements in the intervention literature [13]. When interventions result in weight loss, most also observe improvements in global self-esteem and the competencies most affected, i.e. physical appearance, athletic competence and social acceptance [32]. It is surprising therefore that the degree of weight loss was correlated with self-esteem improvement in only one of the five studies that reported these associations.

We have recently reported on the outcomes of an intensive, residential weight loss programme for youth with obesity. Attendees lost 5.5 kg (−0.25 BMI *z*-score) during an average stay of just over 4 weeks [33•]. Weight loss was positively associated with improvements in athletic competence and physical appearance but not global self-worth. The sample size was large (*N*=303) but the correlation coefficients small (0.13 and 0.19). At the programme start, around one-third had low global self-worth, three quarters had low competence in physical appearance, but less than 17% reported low social acceptance. Only 2.3% (*n* = 7) reported low domain competency across all domains at the beginning of the intervention, and there were none by the end of their stay. By the end of the programme, the proportion with low global self-worth had been reduced to 16%, while those with high global self-worth increased 16.5 to 23%. Most of the improvement in domain competence was in the moderate range of scores, with little change in the number of attendees reporting high scores [33•].

Overall, observations such as these suggest that psychological benefit may be as dependent on some feature of the environment or supportive network as on weight reduction. In the context of group interventions such as residential programmes, these may include the daily company of others who have obesity in common, improvements in competence or self-efficacy in newly prioritised areas (such as exercising regularly), the establishment of new friendships or fewer experiences of weight-related victimisation. These are experienced before adolescents notice levels of weight loss that have either personal or clinical significance [33•].

## Conclusions and Implications

The relationship between obesity and impaired well-being in youth is present but modest in overall strength and varies between individuals. Children with severe and persistent obesity are especially compromised. The ‘myth of psychological maladjustment’ can be dispelled, although the variation in impairment should be recognised.

Consider the key constituents. Psychological features such as low self-esteem are likely minor contributors to the development and maintenance of obesity, albeit with the potential to interact with other risk factors. And obesity is undoubtedly only one of the several influences on an individual’s sense of self-value, albeit a potentially important one. Additionally, both obesity and self-esteem are resistant to change. Longitudinally, any association will be bi-directional, in the same manner to that proposed for the relationship between obesity and depression [34]. Bi-directionality between obesity and impaired health-related quality of life, a concept that overlaps with self-esteem, emerges in middle childhood [35•]. It follows that the pre- to early teenage years is a key period for children in economically developed countries. Changing peer relationships at this age and priorities for physical attractiveness are likely to be critical.

Mood disorders and eating disorders are other markers of impaired well-being, alongside low self-esteem. They are undoubtedly all interrelated. Furthermore, given that obesity persists, then the negativity associated with being fat is likely to accumulate. Unsurprisingly therefore, those who remain obese from early childhood into adolescence have the highest levels of depressive symptoms [36] and binge eating [37]. This is a reminder that the priority for preventing obesity should never distract from addressing the needs of those already obese. For some, these needs are apparent from childhood and continue.

In terms of improving self-esteem, then weight loss is undoubtedly important. But as reflected on above, the child’s environment and supportive networks are also important. As previously observed, many people with obesity, adults and children alike, have high self-esteem, do not suffer major depression, are in well-paid employment and have good social relationships. This implies individual resistance or resilience. Resilience offers a different perspective to the more traditional risk factor approach, focusing on strengths rather than deficits [38•]. It is concordant with an assets-based approach to health improvement that is extremely popular currently in public health. Assets exist within individuals (self-efficacy, drive), close community (family and friends, intergenerational) or are organisational or institutional (housing, representation/advocacy). Identifying and developing assets, many of which are external to the individual, are challenging, especially in an environment rife with anti-fat attitudes. This is consistent with the view that targeting, personalisation and relationships are fundamental to improving the way that young people value themselves [6]. It is also a perspective I am sure that Jane would have supported.
